# Congenital Hypopigmentary Disorders with Multiorgan Impairment: A Case Report and an Overview on Gray Hair Syndromes

**DOI:** 10.3390/medicina55030078

**Published:** 2019-03-25

**Authors:** Laura Cristina Gironi, Francesca Zottarelli, Gianfranco Savoldi, Lucia Dora Notarangelo, Maria Eleonora Basso, Ivana Ferrero, Fabio Timeus, Franca Fagioli, Luigi Maiuri, Enrico Colombo, Paola Savoia

**Affiliations:** 1 Department of Health Sciences, Amedeo Avogadro University of Eastern Piedmont, 28100 Novara, Italy; francesca.zottarelli@gmail.com (F.Z.); luigi.maiuri@med.uniupo.it (L.M.); paola.savoia@med.uniupo.it (P.S.); 2 Department of Pathology, Cytogenetic and Medical Genetics Unit, ASST Spedali Civili, 25123 Brescia, Italy; gianfranco.savoldi@asst-spedalicivili.it; 3 Pediatric Hematology and Oncology Unit, ASST Spedali Civili, 25123 Brescia, Italy; luciadora.notarangelo@asst-spedalicivili.it; 4 Pediatric Onco-Haematology Division, City of Health and Science of Turin, 10126 Turin, Italy; eleonora.basso@unito.it (M.E.B.); ivana.ferrero@unito.it (I.F.); fabio.timeus@unito.it (F.T.); franca.fagioli@unito.it (F.F.); 5 Department of Translational Medicine, Amedeo Avogadro University of Eastern Piedmont, 28100 Novara, Italy; enrico.colombo@med.uniupo.it

**Keywords:** genodermatoses, genetic skin disorders, pigmentation disorders, gray hair syndromes, griscelli syndrome, congenital hypopigmentary disorders

## Abstract

The term congenital hypopigmentary disorders refers to a wide group of heterogeneous hereditary diseases, clinically characterized by inborn pigmentary defects of the iris, hair, and/or skin. They include Gray Hair Syndromes (GHSs), a rare group of autosomal recessive genodermatosis hallmarked by inborn silvery gray hair. GHSs encompass Griscelli, Chediak–Higashi, Elejalde, and Cross syndromes, which are all characterized by a broad spectrum of severe multisystem disorders, including neurological, ocular, skeletal, and immune system impairment. In this manuscript, we describe in detail the clinical, trichoscopic, and genetic features of a rare case of Griscelli syndrome; moreover, we provide an overview of all the GHSs known to date. Our report highlights how an accurate clinical examination with noninvasive methods, like trichoscopy, may play a crucial rule in diagnosis of rare and potentially lethal genetic syndromes such as Griscelli syndrome, in which timely diagnosis and therapy may modify the clinical course, quality of life, and likelihood of survival.

## 1. Introduction

Congenital Pigmentary Disorders (CHD) are a group of heterogeneous, multiorgan diseases, clinically featured by inborn pigmentary defects of the iris, hair, and/or skin and a wide range of neurological, ocular, skeletal, and hematological disorders [1]. These pigmentary defects are congenital and clinically identifiable since the first week of life; therefore, an accurate medical evaluation could provide a rapid, noninvasive, and time-saving diagnostic tool [1,2].

Based on the dermatological characteristics, congenital pigmentary disorders have been classified in gray hair syndromes (GHSs), localized hypopigmentary hair and skin defect syndromes (Waardenburg syndrome and Piebaldism) and generalized hypopigmentary hair and skin defect syndromes (Oculocutaneous Albinism and Tietz syndrome) [1,2,3,4,5,6,7,8,9].

Specifically, the term GHS encloses a rare group of autosomal recessive disorders including Griscelli syndrome (GS), Chediak–Higashi syndrome (CHS), Elejalde syndrome (ES), and Oculocerebral hypopigmentation syndrome, Cross type (OHS). Although GHSs are extremely heterogeneous diseases, they are all clinically hallmarked by inborn silvery gray hairs [5]. Table 1 summarizes the clinical and genetic characteristics of all known GHSs to date [1,2,3,4,5,6,7,8,9,10].

Here we report an uncommon case of GHS in a child who came to our attention for a congenital diffuse hypopigmentation of scalp and body hair. The diagnosis by light microscopy of hair shaft together with the presence of typical dermatological and extracutaneous clinical findings led us to perform the conclusive genetic analysis.

## 2. Case Presentation

**Figure 1:**
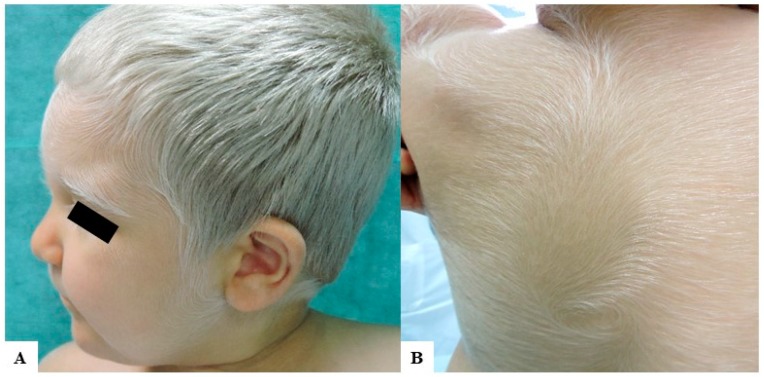
Clinical findings of our patient. (**A**) A 2-year-old Caucasian child with congenital silvery gray scalp hair, eyebrows, and eyelashes and fair skin. He had also brown irises and very fair skin with mild tanning capacities after sun exposure; (**B**) Diffuse silvery hypertrichosis: unmedullated, short, and soft hypopigmented hairs on the back of the patient.

A Caucasian male child came to our attention for inborn generalized hypopigmentation of scalp and body hair. He showed congenital silvery gray hair, eyelashes, and eyebrows (Figure 1A), without neurological and hearing impairment, facial dimorphism, or history of seizures.

He was the first child of apparently healthy parents with second-degree consanguinity. His 30-year-old mother had normally structured blond scalp and body hair. The 37-year-old father presented with the complete absence of hair on the scalp and the body. Up to his 20th year, he had very light blond hair normally distributed on the scalp and body; subsequently, he developed a severe form of alopecia areata that led to alopecia universalis. Both parents were brown-eyed and had fair skin, which can darken after sun exposure.

**Figure 2:**
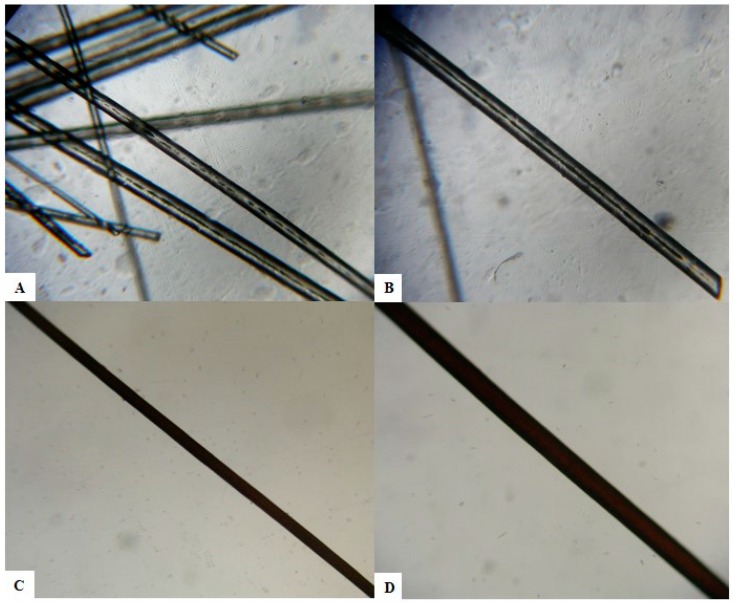
Light microscopic examination of hair shafts of the proband (**A**,**B**) and of a healthy control (**C**,**D**). Giant uneven melanin granules in the medullar zone of the hair’s proband at light microscopy (**A**) 40× magnification; (**B**) 100× magnification. Aspect on light microscopy of a black hair of a healthy Caucasian control. The pigment is homogeneous and evenly distributed in the hair shaft. (**C**) 40× magnification; (**D**) 100× magnification.

Our patient’s history was remarkable also for a diagnosis of hemophagocytic lymphohistiocytosis (HLH), manifested at 3 months of age as an acute severe multiorgan disease with fever, hepatosplenomegaly, pancytopenia, and lymphadenopathy. Chemotherapy and hematopoietic cell transplantation from match unrelated donor were performed with immunological reconstitution. At 2 years of age, the patient was in good condition and assumed oral cyclosporine and corticosteroids. Due to systemic medications, he developed a generalized acquired hypertrichosis involving the entire body hairs, which were silvery, likewise scalp hair, eyelashes, and eyebrows (Figure 1B). Hair light microscopy showed giant uneven melanin granules mostly located approximately in the medullar zone. They were larger than those present in normal hair (Figure 2).

Due to the finding of HLH, the congenital hair and skin defects, and the consanguinity of the parents, a diagnosis of type 2 GS has been suspected.

**Figure 3:**
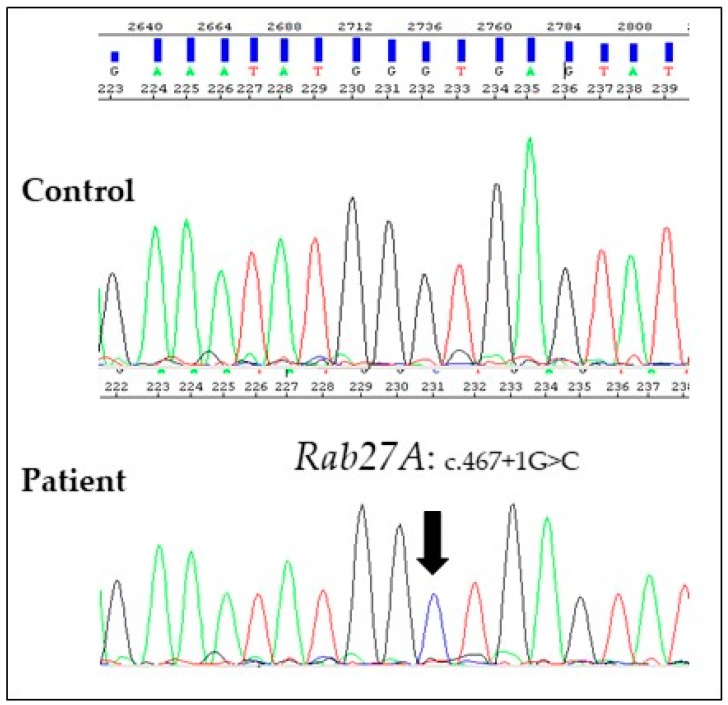
Electropherograms of a control and the affected subject showing the homozygous variant c.467 + 1G > C. Genomic DNA was extracted from bone marrow cells of the proband using an automated nucleic acid purification platform (Maxwell® 16, Promega). The five coding exons of *RAB27A* gene and their flanking intronic regions were amplified by PCR with specific primers (available upon request) and then sequenced by Sanger Sequencing (ABI 3130 Genetic Analyser, Applied Biosistems). The cDNA analysis from total RNA isolated from pretransplantation bone marrow of the proband or from the parents’ peripheral blood sample could define the effect of germline mutations of *RAB27A* gene on its transcription.

After informed consent, we performed genetic analyses on proband’s genomic DNA isolated from bone marrow cells and detected a homozygous mutation in the +1G residue of intron 5 (c.467 + 1G > C) of *RAB27A* gene, confirming our clinical suspicion (Figure 3). The same mutation in a heterozygous state was confirmed in both parents. This mutation affects the invariant splice donor site and is predicted to result in exon skipping or the use of an alternative splice site [11]. Therefore, in accordance with the standards and guidelines of the American College of Medical Genetics (ACMG), we have attributed to this variant a very strong evidence of pathogenicity [12].

## 3. Discussion

Follicular pigmentation depends on melanogenesis, the biochemical process of melanin production, which is regulated genetically at various levels [1,13]. This complex process of synthesis of melanin leads to the wide spectrum of human hair colors; in healthy newborns, it can range from extremely light blond to red or black, while silvery and white hairs represent clinical signs that always require a medical investigation [1,13].

Specifically, silvery hair is a characteristic finding of all GHSs; these are infrequent, autosomal-recessive entities that may also be characterized by immunologic and/or neurologic life-threatening alterations. They encompass Griscelli, Chediak–Higashi, Elejalde and Oculocerebral hypopigmentation (Cross type) syndromes [1,2,3,4,5,6,7,8,9,10].

GS consists of a group of multisystem hereditary disorders featured by CHD, immunological and/or neurological defects [2,3,4]. It was described for the first time at the end of the 1970s; subsequently, three subtypes have been characterized, based on genetic and clinical features. Diffuse congenital skin and hair hypomelanosis with silvery gray hair shaft represent the clinical pathognomonic findings of all subtypes of GS (Table 1) [3,4,5,6]. GS, type 1 (GS1) is caused by mutations in the gene encoding Myosin-VA (*MYO5A*); it is featured by primary neurologic impairment, without immunologic defects. GS, type 2 (GS2), caused by germline mutations in the *RAB27A* gene, is associated with immune dysfunction without primary neurological impairment. *RAB27A* gene encodes a membrane-bound GTPase, RAB27A, involved in protein transport, especially in melanosome transport within melanocytes [3,10]. It is also expressed in T-lymphocytes, in which it plays an important role in granule exocytosis of cytotoxic proteins and in the priming at the immunologic synapse [10]. The pathogenic defect in the *RAB27A* gene is responsible both for inborn pigmentary impairment and for triggering the HLH, a rapidly progressive, life-threatening uncontrolled T-lymphocyte and macrophage activation. GS type 3 (GS3), featured by dermatological congenital abnormalities without neurologic and immunologic defects, is due to germline mutations in the *MLPH* (Melanophilin) gene [5].

Chediak–Higashi syndrome (CHS) is a rare autosomal recessive immunodeficiency disorder characterized by aberrant intracellular protein transport, due to germline mutations in *LYST* lysosomal trafficking regulator gene (also known as *CHS1*). *LYST* mutations cause impairment of lysosomes function in a wide range of cells, such as immune system cells, melanocytes, neurons, and platelets. Therefore, patients with CHS are clinically characterized by recurrent infections, congenital pigmentary disorders, a mild hemorrhagic tendency, and progressive neurologic deterioration; about 85% of them also develop HLH [1,2,7].

Elejalde syndrome (ES), also named neuroectodermal melanolysosomal disease, was first reported at the end of the 1970s in three South American consanguineous families. To date, approximately 20 cases have been reported in the literature, most of which are of Mexican origin. The main clinical features of ES patients are silver-leaden hair, a severe central nervous system dysfunction, and a wide range of ophthalmologic abnormalities; the skin usually shows a generalized hypopigmentation, with ability to tan after sun exposure. The absence of immune dysfunction allows differential diagnosis with GS, type 2. Although the exact genetic basis of ES is still unclear, it has been hypothesized that ES represents an extremely rare variant of GS type 1 caused by *MYO5A* gene mutations [1,2,8].

Oculocerebral hypopigmentation syndrome, Cross type (OHS), is an extremely rare inherited disorder featured by generalized hypopigmentation of the skin, silvery scalp and body hair, and a wide range of central nervous system and eye abnormalities. OHS was first reported in 1967 and, until now, 14 cases have been described, without an ethnic prevalence. Although the gene involved in this syndrome is not yet known, recently Chabchoub et al. suggested that OHS maps to chromosome 3q27.1q29 [11].

## 4. Conclusions

Here we provide an overview on GHSs, a group of extremely heterogeneous life-threatening congenital diseases, in which timely diagnosis can have a crucial impact on the clinical course, the likelihood of survival, and the quality of life. However, due to their rarity and complexity, GHSs can represent a diagnostic challenge for clinicians. Furthermore, the high degree of phenotypical overlap between these disorders and their subtypes makes the identification of the pathogenic mutations mandatory to reach diagnostic certainty.

This report highlights how an accurate dermatological examination could provide a rapid, noninvasive, time-saving diagnostic tool that may help the clinicians to promptly identify which one of the congenital pigmentary disorders to investigative first.

## Figures and Tables

**Table 1:** Clinical and genetic findings of Gray Hair Syndromes.

Name of Syndrome	Clinical Findings		Genetic Findings	Number of Cases Reported Until Now/Epidemiology [11]
	Dermatological Signs	Nondermatological Signs		
GS Griscelli syndrome GS, type 1 (GS1) GS, type 2 (GS2) GS, type 3 (GS3)	GS Hair: Silvery scalp hair, eyebrows, and eyelashes GS Skin: Generalized hypopigmentation with tanning capacities after sun exposure	GS1 Neurologic defects epilepsy, endocranic hypertension, cerebellar signs, hemiparesis, spasticity, hypotonia, peripheral facial palsy, psychomotor retardation Eyes Blue to brown iris	GS1 MYO5A (15q21.2)	GS1 20 cases
		GS2 Immunological defects absence of delayed-type cutaneous hypersensitivity, impaired NK cell function, hypogammaglobulinemia, hemofagocitic lymphohystiocitosis, accelerated phases of hematological disease Neurological defects progressive neurological deterioration due to cerebral macrophage infiltration Eyes Blue to brown iris	GS2 RAB27A (15q21.3)	GS2 102 cases
		GS3 no other systemic signs	GS3 MLPH (2q37.3)	GS3 13 cases
CHS Chediak–Higashi syndrome	CHS Hair: Silvery scalp hair, eyebrows, and eyelashes CHS Skin: Fair skin with tanning capacities (possible hyperpigmentation after sun exposure)	CHS Ocular signs possible strabismus, nystagmus, photophobia Immunological dysfunction recurrent infections (impaired chemotaxis of granulocytes, diminished antibody-dependent cytotoxicity, defective NK cell function); uncontrolled macrophage activation and lymphohistiocytic infiltrates into major organs (about 85% develop HLH) Neurological defects progressive neurological deterioration (cerebral macrophage infiltration) Eyes Blue to brown iris	CHS AR LYST (1q42-43)	600 cases
ES Elejalde syndrome (neuroectodermal melanolysosomal disease)	ES Hair: Leaden-to-silvery scalp hair, eyebrows, and eyelashes ES Skin: Generalized hypopigmentation with intense tanning (bronze skin color) after sun exposure	ES Neurologic defects Like GS1 Ocular signs nystagmus, diplopia, congenital amaurosis Other findings omphalocele	ES AR MYO5A (15q21.2)	20 cases
OHS Oculocerebral hypopigmentation syndrome, Cross type	OHS Hair: Silvery scalp hair, eyebrows, and eyelashes OHS Skin: Generalized hypopigmentation (very light) with photosensitivity	OHS Neurological signs growth deficiency, intellectual disability, spastic tetraplegia, athetoid movements, hyperreflexia Ocular signs microphthalmia, corneal and lens opacity, ectropium, nystagmus, optic nerve atrophy, visual impairment/blindness Other findings Abnormal palate morphology, gingival fibromatosis, dolichocephaly, oligophrenia	OHS AR gene unknown (3q27.1q29)	14 cases
